# Periapical cysts in dogs: 10 cases (2000–2020)

**DOI:** 10.3389/fvets.2024.1493959

**Published:** 2024-12-03

**Authors:** Rebecca J. Vogel-Waski, Ana C. Castejon-Gonzalez, Molly E. Church, Alexander M. Reiter

**Affiliations:** ^1^Red Bank Veterinary Hospital, Dentistry and Oral Surgery Service, Red Bank, NJ, United States; ^2^Department of Clinical Sciences and Advanced Medicine, School of Veterinary Medicine, University of Pennsylvania, Philadelphia, PA, United States

**Keywords:** periapical cyst, radicular cyst, odontogenic cyst, diagnostic imaging, histopathology, dog, canine

## Abstract

**Objective:**

To characterize the clinical, diagnostic imaging, and histologic features with description of treatment outcome of periapical cysts in dogs.

**Animals:**

Ten client-owned dogs diagnosed with periapical cysts biopsied between July 1, 2000 and June 30, 2020.

**Procedures:**

Medical records of the Matthew J. Ryan Veterinary Hospital of the University of Pennsylvania were retrospectively searched to identify dogs that had surgical biopsy specimens of cavitary lesions diagnosed as odontogenic cysts and that met additional inclusion criteria. Complete medical records were reviewed.

**Results:**

Range age and body weight of affected dogs were 2.5–12.1 years and 4.3–38.4 kg (9.5–84.7 lb), respectively. All periapical cysts were affecting the incisive bone and/or the maxilla. Nine dogs presented with a fluctuant swelling of the oral mucosa and gingiva; one dog without clinical swelling presented with a history of difficulty breathing. All cysts originated from a non-vital tooth with a structural defect (wear or fracture without pulp exposure) and/or intrinsic staining. Extraction of the associated non-vital teeth, enucleation of the cysts, and curettage of the surgical sites resulted in resolution of the clinical signs.

**Conclusions and clinical relevance:**

The findings indicate that periapical cysts are associated with a non-vital tooth without pulp exposure. Complete evaluation of the clinical, diagnostic imaging, and histologic features of the lesion in affected dogs is necessary to differentiate periapical cysts from other odontogenic cysts and tumors.

## 1 Introduction

The periapical cyst, also called radicular cyst, is an inflammatory odontogenic lesion arising from proliferation of epithelial cell rests of Malassez around the apex of a non-vital tooth (1–3). Chronic inflammation secondary to pulp necrosis is responsible for proliferation of these cells leading to cyst wall formation and eventual cyst enlargement. The accumulation of cellular debris within the cyst increases the protein content and the osmotic pressure, thus causing transportation of fluid into its lumen and gradual enlargement of the cyst with secondary bone resorption (4, 5). The periapical cyst is classified as pocket cyst if connected with the root canal system or true cyst if not connected (1, 6–9).

Odontogenic cysts are generally categorized into 2 categories: developmental and inflammatory cysts. However, it can be difficult to categorize the cyst in certain cases. Developmental odontogenic cysts include dentigerous cyst, keratinized odontogenic cyst, lateral periodontal cyst, and canine parakeratinized odontogenic cyst. Inflammatory odontogenic cysts include furcation cyst and radicular or periapical cyst (2, 3, 10). Characteristics that differentiate the periapical cyst from other odontogenic cysts include the status of tooth eruption, vitality of the involved tooth, location of the cyst, characteristics of the cyst content, and histopathology of its epithelial lining. A dentigerous cyst includes an unerupted tooth; the lateral periodontal cyst occurs lateral to the root of a tooth and has a thickened epithelium; the furcation cyst develops in the furcation of an erupted vital maxillary fourth premolar tooth; the keratinized odontogenic cyst contains laminated keratin in its lumen and a heavily keratinized wall epithelium; the canine parakeratinized odontogenic cyst is associated with an erupted tooth, and its epithelium is thick and parakeratinized but does not have free keratin in its lumen (2, 10–17).

Options for cyst treatment and removal include removal of the entire structure, including maxillectomy, mandibulectomy or cyst enucleation. Additional ways to remove and kill all or any remaining cystic lining, include electrocautery, CO2 laser, marsupialization and sclerotherapy using materials such as tetracycline and minocycline, types of treatment are often used to remove all visible remnants of the lining, with secondary back up to remove any potential remaining cystic tissue (1, 3, 7, 17).

Some of the diagnostic imaging findings of the periapical cyst are common to other odontogenic cysts as well as periapical granuloma or abscess formation including cortication, round to ovoid lucency, bone resorption, tooth displacement, and root resorption. Therefore, the presence of a periapical radiolucency is not enough to differentiate a periapical cyst from a periapical granuloma/abscess or other odontogenic cysts (10, 17–19). The aim of the present case series is to describe the clinical presentation, diagnosis, and treatment of periapical cysts in dogs.

## 2 Materials and methods

### 2.1 Medical records and inclusion criteria

Medical records of the Matthew J. Ryan Veterinary Hospital of the University of Pennsylvania were searched to identify dogs that had been diagnosed with a cavitary lesion in the tooth associated region of the jaw between July 1, 2000 and June 30, 2020. Search criteria included oral cyst, radicular cyst or periapical cyst, facial swelling, non-vital tooth and uncomplicated crown fracture with cystic lesion. Dogs were included as cases if they had clinical, diagnostic imaging, and histologic evidence of a periapical cyst associated with an erupted, permanent non-vital tooth that was present at the time of the initial diagnosis and did not have a diagnosis of one of the other reported oral cysts including lateral periodontal cysts, keratinized odontogenic cyst or parakeratinized odontogenic cyst. Dogs were excluded if any of the above criteria were not met or the clinical, diagnostic imaging, or histologic information was insufficient or not available for review.

### 2.2 Clinical and diagnostic imaging information

The signalment of the dog (breed, gender, weight, and age), medical history, presenting complaint, clinical signs, and clinical oral and diagnostic imaging findings were retrieved from the medical records. Clinical photographs and dental radiographs were evaluated by board-certified veterinary dentists, and computed tomography (CT) images were reviewed by board-certified veterinary radiologists when available. The origin (subject tooth), fluctuance, size, extent, and content of the cyst, the structural and endodontic health of the subject tooth, and the presence of bone loss, corticated borders, displacement and/or resorption of teeth around the cyst were recorded. Any intrinsic staining, wear or fracture of the subject tooth was recorded. Evidence of dental wear included the presence of a smooth wear facet with a yellow to brown stain in its center without pulp exposure consistent with tertiary dentin. Signs of non-vitality of the subject tooth included cessation of dentin production including failure of the pulp chamber to narrow and/or presence of periapical pathology (rarefying or sclerosing osteitis). Specific attenuation features on CT (e.g., contrast enhancing rim surrounding a non-enhancing core) were determined.

### 2.3 Culture, cytologic, and histologic information

Culture, cytologic, and histologic results were retrieved from the medical records. Cytologic smears from aspirated fluid and microscopic sections from tissue samples (prepared by conventional methods, fixed in neutral-buffered 10% formalin, decalcified in a standard 10% HCl solution as needed, embedded in paraffin, sectioned at a 5-μm thickness, and stained with hematoxylin and eosin [H&E]) were examined by board-certified veterinary pathologists.

### 2.4 Treatment and outcome

The extent of tooth extraction (subject teeth and additional adjacent teeth), cyst enucleation, use of electrocautery, laser or sclerotherapy including tetracycline or minocycline, postoperative pain medication and antibiotics, healing progress at recheck examinations, and duration of longest follow-up were assessed.

### 2.5 Data analysis

Continuous data were reported as mean, median, and range. Categorical variables were reported as numbers and percentages.

## 3 Results

Ten dogs were included in the study. There were four female-spayed and six male-castrated dogs. Mean age was 6.9 years (median, 6.7 years; range, 2.5–12.1 years), and mean body weight was 16.9 kg (37.3 lb.; median, 11.8 kg [26.0 lb]; range, 4.3–38.4 kg [9.5–84.7 lb]). The breeds included two papillons, one pug, one beagle, one dachshund, one mixed breed, one American pitbull terrier, one standard poodle, and two boxers (Table 1).

**Table 1:** Clinical, diagnostic imaging, culture, cytologic, histologic, treatment and outcome information of 10 dogs with periapical cysts.

Breed, gender	Weight (kg), age (yr)	Duration and location of facial swelling	Nasal signs	Ocular signs	ST*	Defect at ST	Discolored ST	Vital ST	TR	Tooth displacement	Corticated borders	Cyst extension*	CT	Procedure	Nature of cyst fluid	Culture	Cytology	Biopsy	Longest follow-up (days)
PAPS, MC	4.3 kg, 7.1 yr	2 weeks; incisive region/ rostral maxilla	Y	N	201	N	Y	N	N	Y	Y	201–204; medially, laterally	N	XSS, EN	NR	*Pasteurella multocida*	N	Y	106
PAPS, MC	6.8 kg, 8.6 yr	1.5 months; incisive region/ rostral maxilla	Y	N	202	N	Y	N	N	Y	Y	201–208; medially, laterally	N	XSS, EN	NR	N	N	Y	18
PUGS, FS	8 kg, 3.8 yr	No facial swelling, but 1-year history of difficulty breathing	Y	N	203	Y	N	N	N	Y	Y	203 to missing 208 (and into nasopharyngeal meatus); medially	Y	XSS, EN, sclerotherapy (tetracycline injected prior to EN; removed after 10 min)	Dark	N	Y	Y	14
BEAG, FS	8.9 kg, 7.8 yr	1 year; caudal maxilla	N	N	109	Y	N	N	N	Y	Y	108–110; laterally, retroorbitally	Y	XSS, EN, electrocautery	NR	Y (negative)	N	Y	NR
DACH, MC	9.4 kg, 11 yr	1 year; middle maxilla	Y	Y	206	Y	N	N	N	Y	Y	204 to missing 208; medially, laterally	N	XSS, EN, laser	NR	Y (negative)	N	Y	19
MIXB, MC	14.2 kg, 2.5 yr	7 months; middle maxilla	N	Y	204	Y	N	N	N	y	Y	204–208; medially, laterally, dorsally	Y	XSS, EN	Brown-red	N	N	Y	14
APBT, FS	25 kg, 12 yr	3 months; caudal maxilla	N	N	207	N	Y	N	N	N	Y	206–209; medially, laterally	Y	XSS, EN	NR	N	Y	Y	25
SPOO, MC	26 kg, 4.9 yr	Unknown (accidental finding); incisive region/ rostral maxilla	N	N	203	Y	N	N	N	Y	Y	203–204; medially, laterally	N	XSS, EN, defect filled with bioglass	NR	N	Y	Y	98
BOXE, MC	28 kg, 4.1 yr	2 months; middle maxilla	N	N	206	N	Y	N	N	N	Y	205–208; medially, laterally	N	XSS, EN, sclerotherapy (minocycline)	Muddy brown	N	N	Y	48 (some wound dehiscence at 21 days)
BOXE, FS	38.4 kg, 6.2 yr	1 months; caudal maxilla	N	N	107	N	Y	N	Y	N	Y	106–108; medially (minimal), laterally	Y	XSS, EN	NR	N	N	Y	13

PAPS, Papillion; PUGS, Pug; BEAG, Beagle; DACH, Dachshund; MIXB, Mixed breed; APBT, American pitbull terrier; SPOO, Standard poodle; BOXE, Boxer; MC, Male castrated; FS, Female spayed; Y, Yes or present; N, No or not present; ST, Subject tooth; *, Triadan tooth number; TR, Tooth resorption; CT, Computed tomography; XSS, Open (with flap) tooth extraction of ST ± other affected teeth; EN, Cyst enucleation; NR, Not reported.

Medical history and presenting complaint included the presence of a unilateral facial swelling in nine dogs (2 weeks to 1 year duration in eight dogs and no known duration in one dog). One dog had no history of facial swelling, but the owner reported difficulty in breathing with unilateral nasal discharge, and occasional sneezing and epistaxis for 1 year. Prior to referral, nine dogs received an antibiotic (clindamycin, cefpodoxime proxetil, amoxicillin-clavulanic acid, and/or enrofloxacin), four dogs a non-steroidal anti-inflammatory drug (carprofen, deracoxib, and/or meloxicam), and two dogs a glucocorticoid (prednisone), but no significant improvement of the facial swellings or respiratory signs was noted.

All periapical cysts were in the incisive and maxillary bones: incisive bone/rostral maxilla (teeth 01–04) in three dogs, middle maxilla (teeth 05–07) in three dogs, and caudal maxilla (teeth 08–10) in three dogs. The dog with difficulty breathing had all three regions involved. Nasal signs were present in four dogs and included sneezing (*n* = 4), epistaxis (*n* = 2), upper respiratory noise (*n* = 2), nasal discharge (*n* = 1), and decreased nasal airflow with unilateral complete obstruction (*n* = 1); the clinically visible swellings in dogs with nasal signs were in either the incisive bone/rostral maxilla or middle maxilla. Ocular signs (ipsilateral ocular discharge) were present in two dogs with swellings in the rostral maxilla. Nine dogs were eating and drinking well at the time of presentation (eight without pain, one was rubbing its face). Exercise intolerance was noted in one dog due to difficulty breathing through its nose, but it ate and drank well without pain. One other dog ate and drank well until hitting its head 1 week earlier, upon which the swelling in the rostral maxilla was noted by the referring veterinarian as an incidental finding unrelated to the recent trauma.

**Figure 1:**
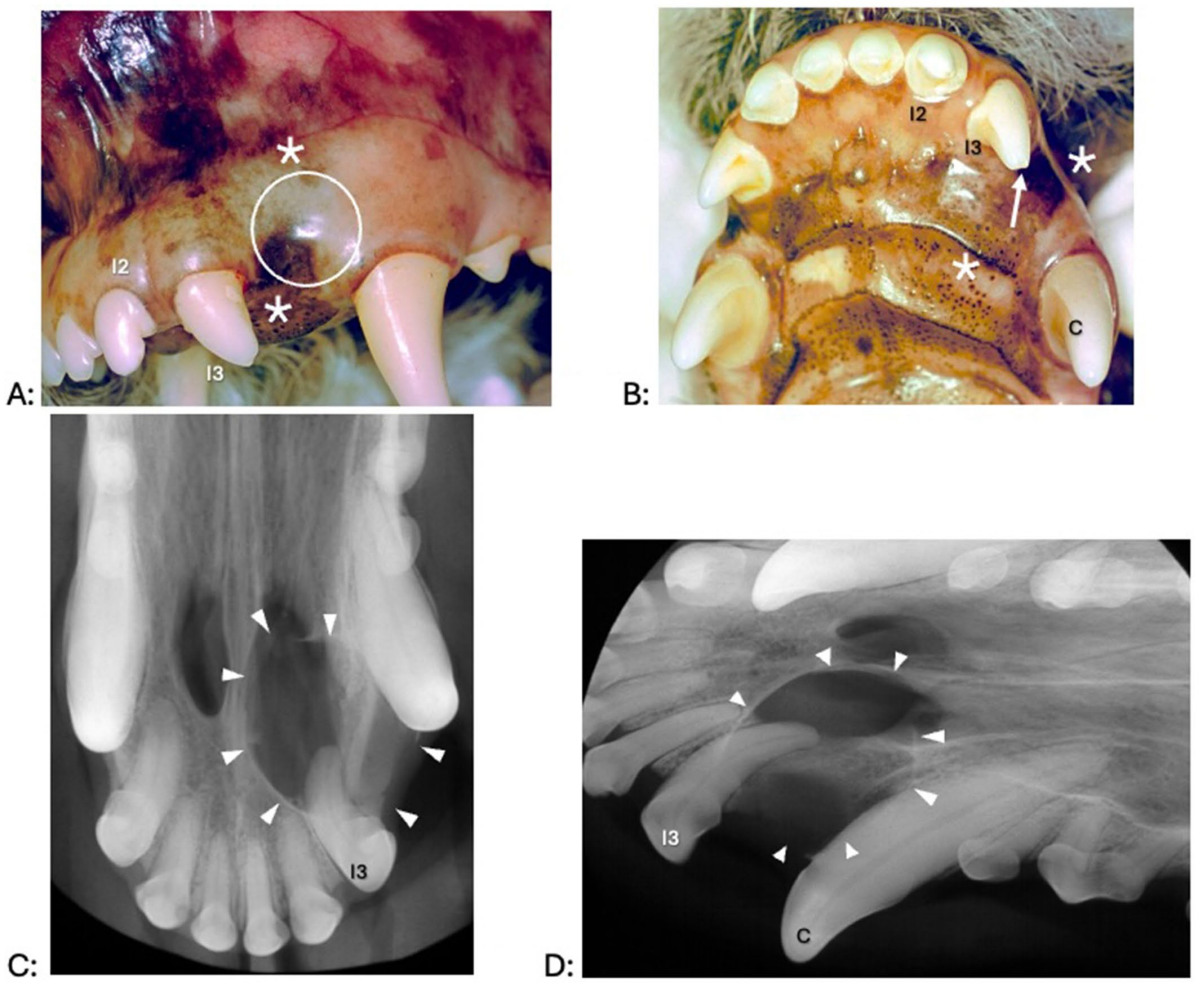
Clinical photographs (A,B) and dental radiographs (C,D) of a 4.9-year-old standard poodle with facial swelling of unknown duration noted on physical examination 1 week earlier. (A,B) The left maxillary third incisor tooth shows an enamel fracture without pulp exposure (arrow). Interdental spaces are increased between the left maxillary second and third incisor tooth and left maxillary third incisor and canine teeth. A fluid-filled swelling (asterisk) expands palatally and labially; there is a bluish discoloration of the gingiva (dotted circle). (C,D) An area with geographic bone loss with corticated borders (arrowheads) is associated with the left maxillary third incisor tooth.

**Figure 2:**
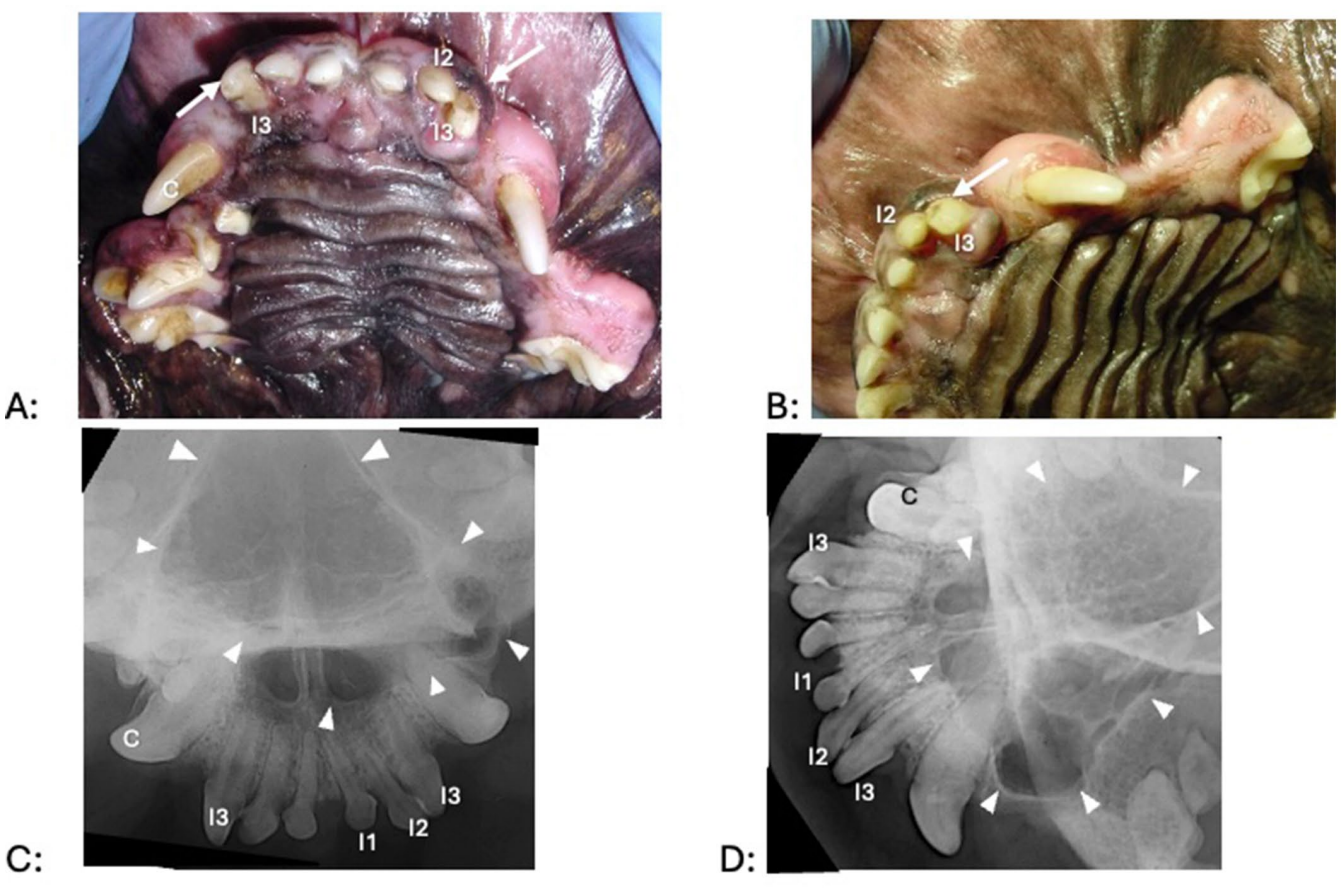
Clinical photographs (A,B) and dental radiographs (C,D) of a 3.8-year-old pug with a 1-year history of difficulty breathing, left nasal discharge (clear to yellow), occasional sneezing and epistaxis, and no appreciable left-sided airflow on physical examination. (A,B) No obvious facial swelling is visible on clinical examination. There is mild to moderate periodontal disease and gingival enlargement. The left premolar teeth are clinically missing. All incisor teeth exhibit mild tooth wear, but a structural defect without pulp exposure (arrow) is most obvious at the left and right maxillary third incisor teeth. The right maxillary third incisor, right maxillary canine, left maxillary second incisor and left maxillary third incisor teeth appeared discolored. (C,D) Third incisor, right maxillary canine, left maxillary second incisor and left maxillary third incisor teeth show a failure to narrow of the pulp chamber, consistent with being non-vital. The integrity of the palatine fissures is lost, and nasal turbinates are missing. The white arrowheads outline the approximate extension of radiographic abnormalities.

**Figure 3:**
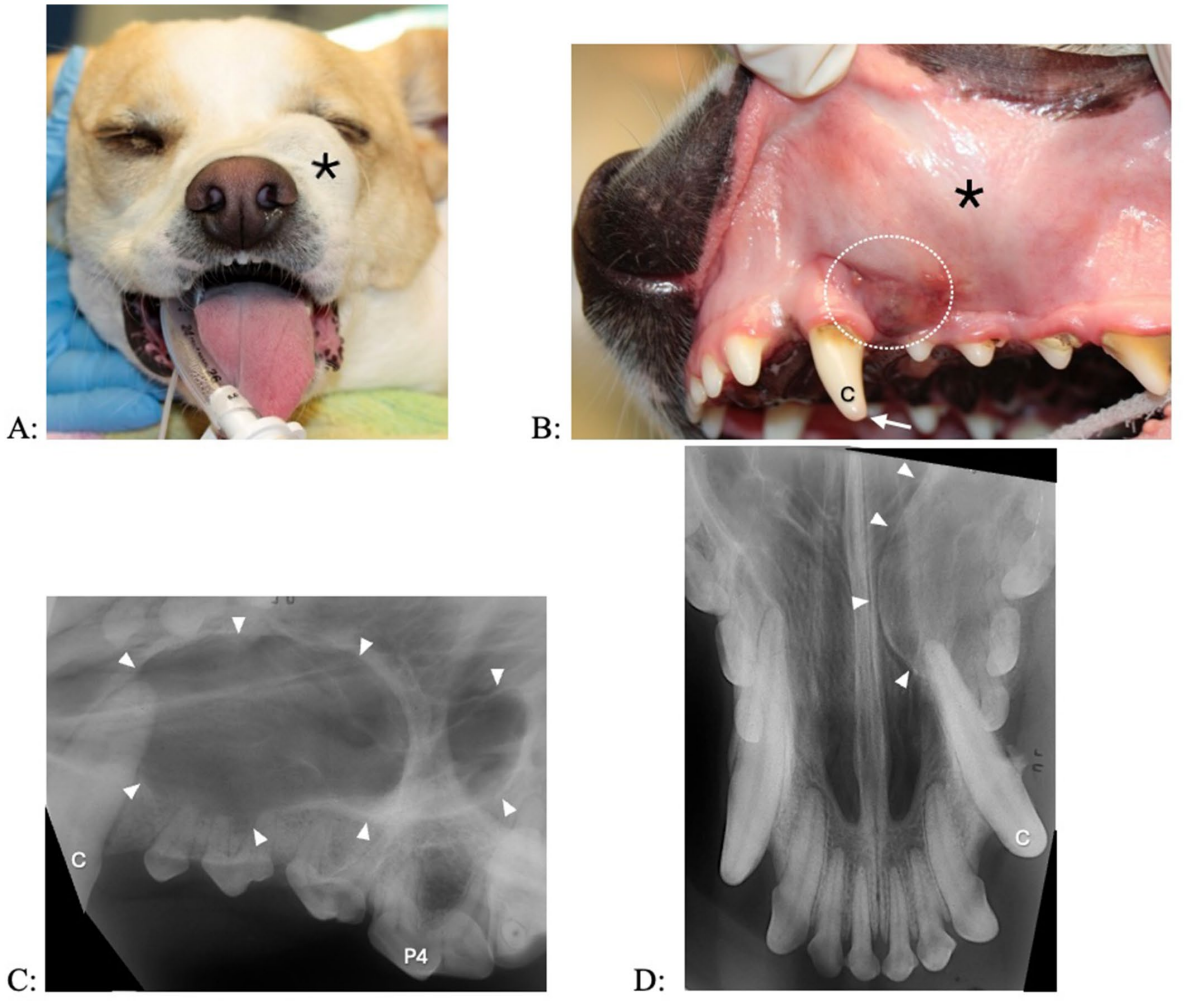
Clinical photographs (A,B) and dental radiographs (C,D) of a 2.5-year-old mixed breed dog with a 7-month history of facial swelling. (A,B) A fluid-filled swelling (asterisk) expands labially and dorsally. The left maxillary canine tooth shows a structural defect without pulp exposure (arrow); mild intrinsic staining is present at the coronal most aspect of the tooth. There is some irritation in gingiva and alveolar mucosa (dotted circle) distal to the left maxillary canine tooth, where the referring veterinarian previously had aspirated serosanguineous (brown to red) fluid. (C,D) A large periapical lucency with corticated borders (arrowheads) expands from the left maxillary canine tooth to the fourth premolar tooth.

**Figure 4:**
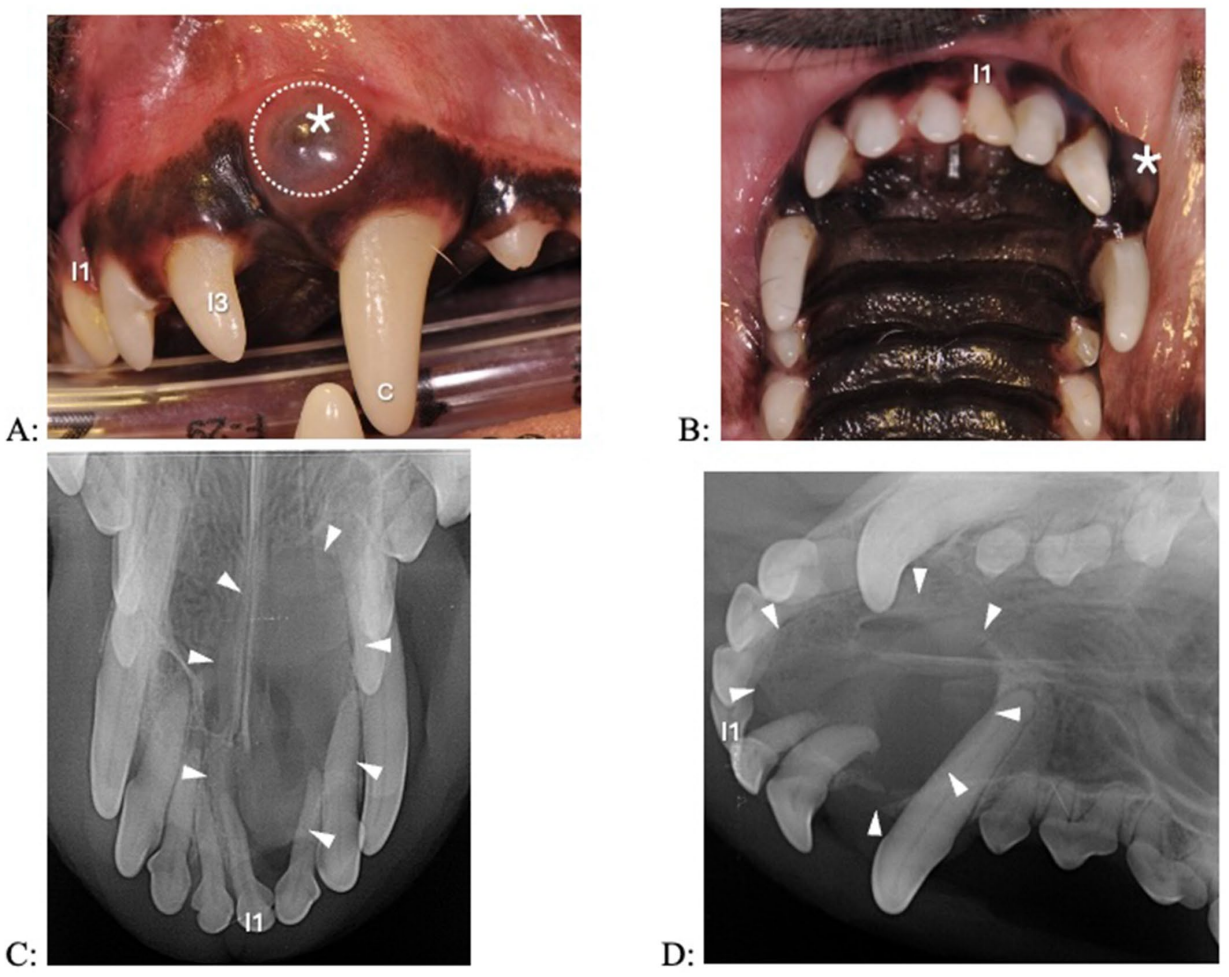
Clinical photographs (A,B) and dental radiographs (C,D) of a 7.1-year-old papillon with a 2-week history of facial swelling. (A,B) The left maxillary first incisor tooth appears discolored and slightly displaced. A fluid-filled swelling (asterisk) expands laterally; there is a bluish discoloration of the gingiva (dotted circle). (C,D) A large periapical lucency with corticated borders (arrowheads) is associated with the left incisive/rostral maxillary region, causing displacement of multiple incisor teeth. The left maxillary first incisor tooth has a wider root canal width compared to the other incisor teeth, indicating that it is non-vital.

A fluid-filled swelling could be seen and palpated underneath gingiva, alveolar mucosa, and/or labial/buccal mucosa in nine dogs (having a bluish hue in two dogs where the cyst was located underneath gingiva only) (Figure 1). An obvious swelling could not be appreciated in the one dog with a history of difficulty breathing (Figure 2). Incisor teeth were considered the cyst origin in four dogs, the canine tooth in one dog, 2nd or 3rd premolar teeth in four dogs, and the first molar tooth in one dog. A small structural defect (wear or fracture without pulp exposure) was present at the crown of the subject tooth (uncomplicated fracture or abrasion) in five dogs (Figure 3). The subject tooth of the other five dogs was intact but showed subtle intrinsic staining (Figure 4). Displacement of subject or adjacent teeth was noted in seven dogs. Beyond gingivitis or mild periodontitis, there were no signs of moderate to severe periodontal disease or sinus tracts at the subject and/or adjacent teeth.

**Figure 5:**
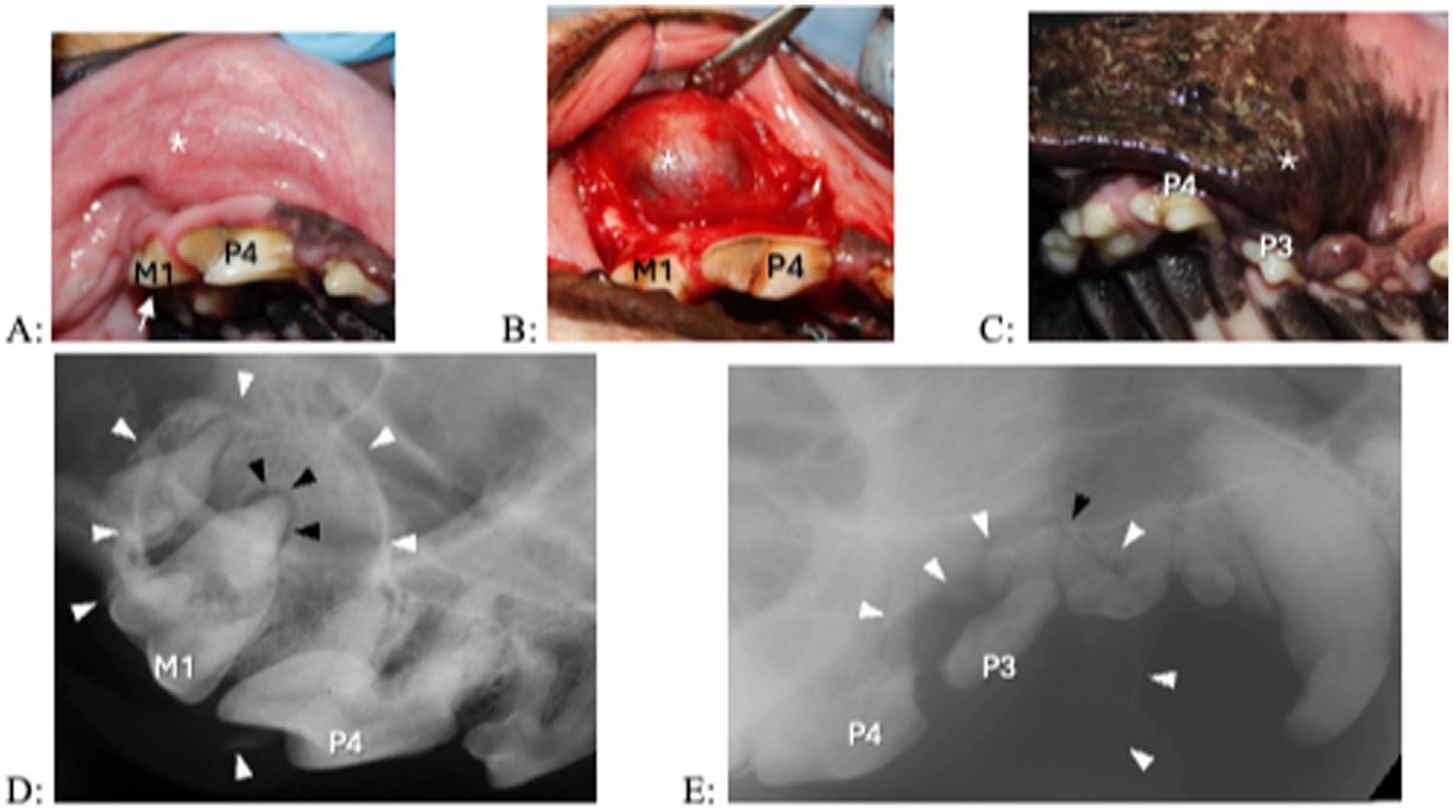
Clinical photographs (A–C) and dental radiograph (D,E) of from 2 dogs with a 1- year history of facial swelling. (A,B) A fluid-filled swelling (asterisk) expands laterally above the right maxillary fourth premolar and first molar teeth. The right maxillary first molar tooth shows a subtle structural defect without pulp exposure (arrow). Upon raising a full thickness mucoperiosteal flap, the outer cyst lining becomes visible. (C) A fluid-filled, laterally expanding swelling (asterisk) is located over the right maxillary premolar region. The right maxillary third premolar tooth appears discolored. (D,E) A large lesion with corticated borders (white arrowheads) is centered at the right maxillary first molar tooth and centered over the right maxillary third premolar tooth. In (D) the black arrowhead indicates periapical lucency at the palatal root of the right maxillary first molar tooth, in (E) the black arrowhead indicates periapical lucency at the mesial root of the right maxillary third premolar tooth. In that patient, resorption is also present in the distal root at the right maxillary third premolar tooth and the mesial roots at the right maxillary fourth premolar tooth.

**Figure 6:**
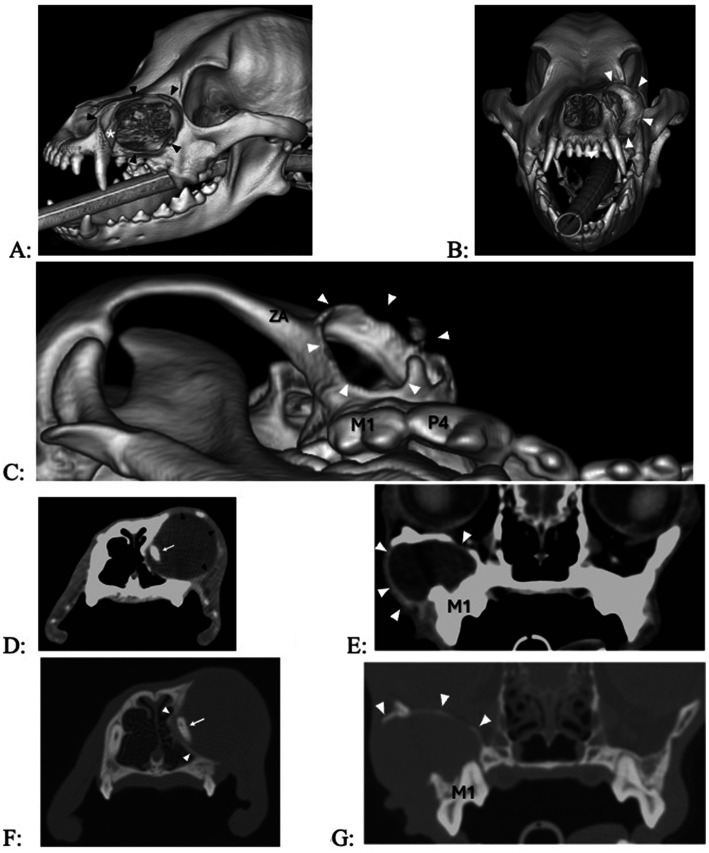
(A–C) 3D renderings of two dogs with a 7- and 12-month history of facial swelling, showing extensive bone loss at the maxilla (A,B) and the rostral aspect of the zygomatic arch (C). (D,E) Transverse images in soft tissue algorithm show a smooth-margined, expansile, lytic mass in two dogs with signs of exophytic, endophytic, and expansile changes into the nasal passages (D) and retroorbital space (E), contrast-enhancing borders in the lateral aspect of the lesion [black arrow heads in (D), white arrowheads in (E)], and a fluid-attenuating center. (F,G) Transverse images in bone algorithm show outward displacement and thinning/disappearance of the cortices.

**Figure 7:**
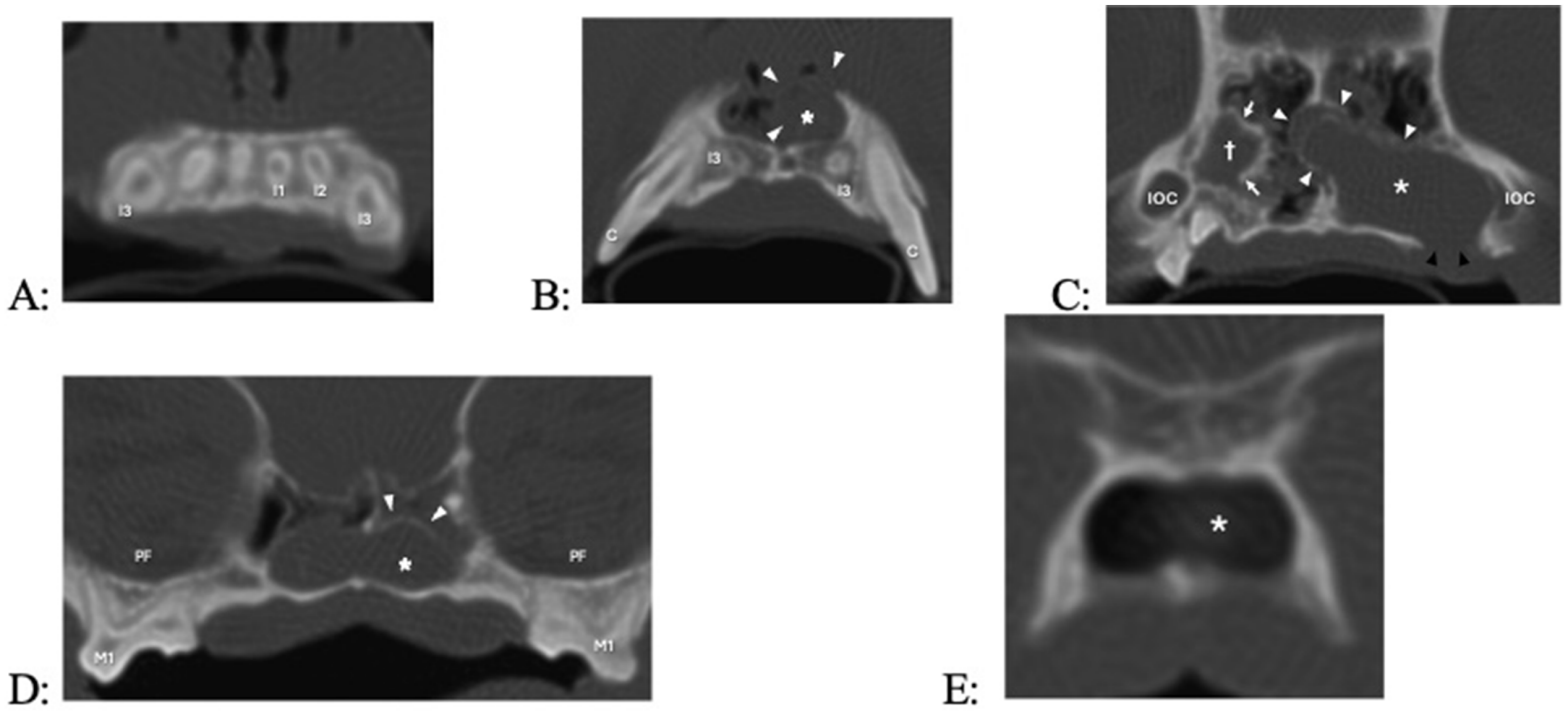
Transverse computed tomography (CT) images in bone algorithm of a 3.8-year-old pug with a 1-year history of difficulty breathing, left nasal discharge (clear to yellow), occasional sneezing and epistaxis, and no appreciable left-sided airflow on physical examination. There is a left expansile nasal mass with associated bone destruction (primarily of the left maxilla and nasal turbinates), displacement of the nasal septum to the right (causing the right nasal passage to be decreased in size), and fluid attenuatingn material occupying most of the caudal nasal cavity, and occlusion of the nasopharyngeal meatus and nasopharynx. There are two cyst-like structures, the smaller one originating from the right maxillary canine tooth (not counted, as there was no biopsy performed) and the larger one originating from the left maxillary third incisor tooth. (A) Level of the roots of the maxillary incisors (the root canal width of right maxillary first incisor, left maxillary first second and third incisor teeth failed to narrow, indicating that they are non-vital). (B) Level of the apices of left and right maxillary third incisor teeth, with cyst (asterisk, white arrow heads) originating from the left maxillary third incisor tooth. (C) Level of the infraorbital canals (IOC), with a cyst-like structure at the right maxillary canine tooth appearing (cross, white arrows), the cyst of the left maxillary third incisor tooth further enlarging, and bone loss at missing left maxillary third and fourth premolar teeth which had previously been extracted (black arrowheads). (D) Level of the left and right maxillary first and second molar teeth and pterygopalatine fossae (PF); the cyst-like structure originating from the right maxillary canine tooth is no longer visible, and the cyst originating from the left maxillary second incisor tooth has crossed to the other side. (E) Level of the transition between the nasopharyngeal meatus and nasopharynx, with the cyst fading away.

Dental radiography was performed in all dogs, and five dogs also had CT with pre- and post-contrast studies of the head. The subject tooth was determined to be non-vital in all dogs based on diagnostic imaging findings of either failure of the pulp chamber to narrow (relatively wide root canal space) and/or presence of periapical pathology (Figure 5). Resorption of subject teeth or teeth adjacent to them was present in one dog (based on dental radiography and CT) (Figure 5). Tooth resorption was not noted in nine dogs (five based on dental radiography and four based on dental radiography and CT). Reported findings in the five dogs with CT included the presence of an expansile lesion with associated bone destruction, thinning cortices, fluid attenuating center, and post-contrast rim enhancement (Figures 6, 7). The cyst extended dorsomedial into the nasal passages in one dog; dorsomedial into the nasal passages and laterally in seven dogs; dorsomedial into the nasal passages, laterally and dorsally in one dog; and laterally and into the retrobulbar space in one dog. Corticated borders surrounding a radiolucency were seen in all 10 dogs. A second periapical cyst-like structure was found in the patient with a history of difficulty breathing; however, this tooth was not included in the study due to lack of complete diagnostics (Figure 7).

All dogs received regional analgesia (nerve blocks) prior to surgical tooth extraction and cyst enucleation. Large mucoperiosteal flaps were made and sutured closed in two layers (connective tissue and oral mucosa). Subject and adjacent teeth were extracted in nine dogs; one dog had only its subject tooth extracted. Adjacent teeth were removed due to loss of alveolar bone, root resorption, or to allow more complete access to the cyst and cyst enucleation. The color of the cyst fluid was described as brown-red in three dogs and not reported in seven dogs. Electrocautery was used in one dog, CO_2_ laser in one dog, and sclerotherapy in two dogs (tetracycline injected into the cyst and left for 10 min prior to cyst enucleation in one dog; minocycline applied into the cyst cavity post enucleation in another dog) out of concern for residual cyst lining remaining after enucleation. The defect was filled with bioglass in one dog following enucleation to assist in focal wound healing and bone remodeling.

**Figure 8:**
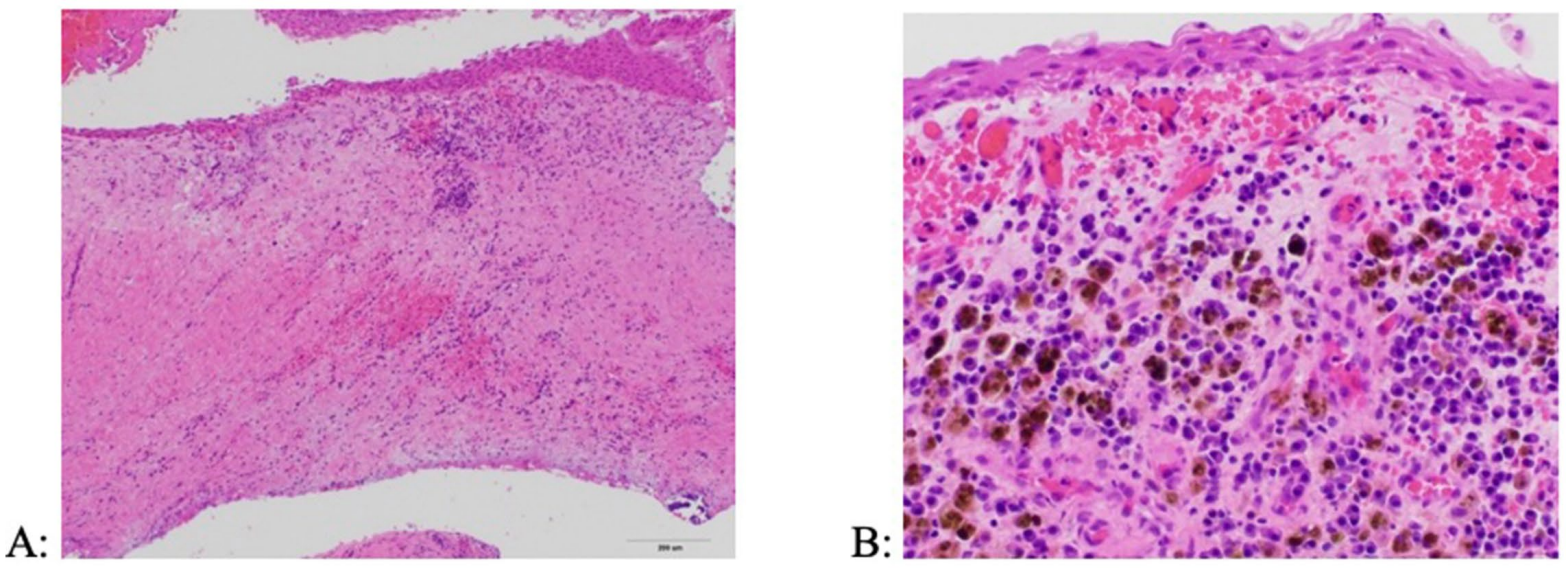
Histologic appearance of a periapical (radicular) cyst. (A) The cyst wall is composed of a fibrous connective tissue stroma lined by a stratified squamous epithelium and containing inflammatory infiltrates (H&E, 10x). (B) Inflammation is primarily comprised of lymphocytes, plasma cells, and hemosiderin-laden macrophages (H&E, 40x).

Culture of the cyst fluid were performed in three dogs (all performed by the referring veterinarian), of which two were negative and one showing *Pasteurella multocida*. Cytological evaluation was performed in three dogs, showing chronic neutrophilic inflammation with evidence of chronic hemorrhage (neutrophils, rare squamous epithelial cells, hemosiderin-laden macrophages, free hemosiderin, but no microorganisms). Histologic evaluation from tissue samples in all dogs revealed the presence of a non-keratinizing stratified squamous epithelium and a subjacent wall comprised of granulation tissue and mature fibrosis with an inflammatory infiltrate characterized by lymphocytes, plasma cells and hemosiderin-laden macrophages (Figure 8). Some cysts had foci of mineralization as well as woven bone proliferation within the cyst wall.

Postoperative pain medication (tramadol, fentanyl patch, gabapentin, carprofen, meloxicam) was prescribed in nine dogs, systemic antibiotics (amoxicillin/clavulanic acid, clindamycin) in six dogs, and glucocorticoids (prednisone) in two dogs. There was no follow-up information in one dog. The first recheck examination for the other nine dogs occurred in 13–25 days. The wound was healing or had healed well in eight dogs. One dog with minocycline applied into the cyst cavity post cyst enucleation showed minor wound dehiscence, which was left to heal by second intention. Mean longest follow-up time was 39.4 days (median, 19 days; range, 13–106 days), with all surgical sites healing or having healed well without recurrent swelling or other clinical signs. Repeat imaging was not performed in any of the included patients to confirm resolution of radiographic signs.

## 4 Discussion

The periapical cyst is the most common oral cyst diagnosed in people followed by the dentigerous cyst (8, 19). In dogs, dentigerous cysts are most common, and periapical cysts are rarely reported (7, 11, 16, 20–23). Up to 64% of the odontogenic cysts diagnosed in people are periapical cysts (8, 24, 25), whereas in dogs only one study reported a prevalence of 2.4% (10). The results of the present study confirm that periapical cysts are rarely diagnosed in dogs, as only 10 cases fulfilled the inclusion criteria during a 20-year period at a dentistry and oral surgery service in a multi-specialty referral hospital. This discrepancy between people and dogs may be due to various reasons. Oral examinations and dental radiography are much more frequently performed on people than dogs. The cysts in the present study were chronic and large enough to cause clinical signs. Small periapical cysts may be misdiagnosed as periapical granulomas, which usually resolve after tooth extraction or endodontic treatment. Inclusion criteria in the present study required confirmed histopathology in addition to clinical and diagnostic imaging evidence of a periapical cyst associated with an erupted, permanent non-vital tooth. Any cases outside these search criteria were excluded, which may have skewed the data to a smaller population of dogs than may otherwise have been found.

Periapical cysts are secondary to inflammatory changes at the apex of the tooth associated with non-vitality (1, 2). Human literature references chronic pulpitis, including in patients with previous endodontic treatment, as a finding associated with periapical cysts. In other articles, the vitality of the tooth prior to treatment was not directly described (1, 5, 24). Veterinary references have noted non-vital teeth for dogs with periapical cysts (2, 11). It is possible that there is a subset of patients with periapical cysts that have chronic pulpitis where the tooth is still vital at the time of diagnosis, as was found in a study about furcation cysts in dogs (10). The non-vitality of a tooth associated with a periapical cyst in the present study was diagnosed in all cases by diagnostic imaging (dental radiography and/or CT) with failure to narrow of the pulp chamber. The patients also had signs of small structural defects (abrasion or uncomplicated fracture) or intrinsic staining on clinical examination at each tooth included in the current study. The human literature finds carious lesions and failed root canal therapy to be the most common causes of periapical cysts secondary to periodontitis (9, 26). It has previously been demonstrated that nearly 25% of teeth with uncomplicated crown fracture exhibited periapical lucencies (“lesions of endodontic origin, LEO) (18, 19), and about 90% of discolored teeth had been found to be non-vital (27, 28). However, we do not know how many of those non-vital teeth have a periapical granuloma or cyst. In humans, less than 20% of periapical granulomas will become cysts due to stimulation of the epithelial cell rests of Malassez (29–31). If the same were to happen in dogs, some periapical cysts may be misdiagnosed as periapical granulomas.

In humans, anterior maxillary teeth were reported to be most affected by periapical cysts, followed by posterior maxillary teeth and anterior mandibular teeth (8). The periapical cysts of dogs in this case series affected maxillary teeth only and were equally distributed between rostral, middle, and caudal locations. Based on the current study and review of the veterinary literature, most cysts were diagnosed in the rostral maxilla, with one case report describing a periapical cyst in the rostral mandible (7, 11, 20–23). In one veterinary study, concussive injuries were found to occur more often in incisor and canine teeth in dogs due to their exposure to external trauma (32). Injuries, such as concussive injury, can result in intrinsic staining due to pulpal hemorrhage and subsequent pulp necrosis and periapical disease. However, because of the low number of cases in the present study and the likelihood of underdiagnosing small periapical cysts or misdiagnosing them as periapical granulomas, it is difficult to determine whether there is a true predilection toward the upper jaw over the lower jaw for development of periapical cysts in dogs.

Dogs with odontogenic cysts generally present with a non-painful swelling of the tooth-bearing region of the jaw, or the cyst is an incidental finding during the routine oral examination (7, 10, 14, 20–23). In the present study one of the 10 dogs presented with discomfort (i.e., pawing at the face). Upper respiratory signs (such as sneezing with or without nasal discharge) were present in 40% of the dogs. If periapical cysts are large enough, they can cause partial or complete upper airway obstruction (33) which was noticed in two dogs of the present study. Therefore, odontogenic cysts should be included as a differential diagnosis in dogs with maxillary swelling and upper respiratory signs. The fluctuant swelling of a periapical cyst that is visible on oral examination may be only a smaller part of the lesion and not be centered on the non-vital subject tooth. If the cyst expands deeply into the nasal cavity, it may become difficult to enucleate (remove the cyst in one piece) or remove all its cyst lining by curettage. Appropriate advanced diagnostic imaging including conventional computed tomography with soft tissue and bone algorithm and/or cone beam CT imaging are necessary to evaluate to the true extent of the cystic lesion (3, 9, 34–36).

Small periapical lucencies are difficult to attribute to a particular periapical disease, but a lucency larger than 2 cm is assumed to be a cyst in humans (1, 9). While larger periapical lesions were predominantly determined to be periapical cysts, some histologically diagnosed cysts manifested radiographically as lesions smaller than 6 mm (34, 35, 37). Differentiation between a periapical cyst and a periapical granuloma may be impossible with dental radiography and without histopathology (34, 38). Cone-beam CT (CBCT) has been shown to be more sensitive than dental radiography (91% vs. 73%) (34). This type of imaging is able to show a 3-dimensional view of the tooth and bone in the area, whereas dental radiography is assessing a 2-dimensional view of a 3 dimensional structure. Human and veterinary dental specialists have begun to shift primarily to CBCT imaging for boney lesions and even primary dental hygiene assessment. Ultrasonography with doppler and texture analysis of CBCT images shows promising results in correctly differentiating periapical lesions in humans (34, 37). Because some periapical cysts in the dogs of the present study were very large, neoplasia (such as ameloblastoma with cystic manifestation) should also be considered as a differential diagnosis. In the authors opinion computed tomography or CBCT is the most appropriate type of diagnostic imaging available to evaluate lesion extension and improve surgical planning. When available, post contrast soft tissue CT studies can be exceptionally helpful in further planning and diagnosis in a cyst and cyst like mass.

Immunohistochemistry has been utilized in humans to differentiate between periapical granulomas, odontogenic cysts, and odontogenic tumors (4, 39–45). There is extensive research with inflammatory markers (e.g., Galectin 1 and 7, intercellular adhesion molecular-1, degranulated mast cells, TGFb, IFNd, TNFa, IL4, IL6), observing differences in expression between periapical cysts and periapical granulomas (36–40). Expression of other markers related to bone resorption (cathepsin, RANKL, OPG) and cellular proliferation (Ki67) is different between odontogenic cysts and ameloblastoma or odontogenic keratocysts (4, 42). Some of the marker are specific to humans, and similar marker may not be available or have not been validated for dogs. In this study, immunohistochemistry was not utilized, but it could be a helpful diagnostic tool if the normal diagnostic tools are not able to diagnose completely.

In the current study, histopathologic evaluation revealed the presence of non-keratinizing stratified squamous epithelium, granulation tissue with lymphocytes, plasma cells and hemosiderin-laden macrophages, and areas of woven bone proliferation. Periapical cysts initially form at the root apex of a tooth and are associated with inflammation and often exhibit bone resorption (2, 7, 10, 11, 22, 33). While an epithelial lining is present in dentigerous, furcation, and periapical cysts, the presence of subepithelial inflammation is key to distinguishing periapical cysts from other types of cysts as is the presence of an associated non-vital tooth. Dentigerous cysts are associated with vital, unerupted teeth with the cyst initially forming at the crown of the tooth (14–16), whereas furcation cysts are associated with vital maxillary fourth premolar teeth in dogs, with the cyst forming at the furcation region of the tooth (10).

Cultures were performed in three dogs included in the present study; two showed no growth, and one grew *P. multocida*. This microorganism is a gram-negative, non-motile penicillin sensitive bacterium that is often considered a core oral bacterium in dogs and cats that can be easily transmitted to humans and other animal species (46, 47). The presence of *P. multocida* was most likely a contaminant as opposed to being a primary bacterium associated with the formation of the periapical cyst.

Fluid analysis and cytology was performed on three dogs of the present study. Fluid analysis revealed serosanguinous material with dark to muddy brown-red appearance. The cytology showed chronic neutrophilic inflammation with evidence of chronic hemorrhage including neutrophils, rare squamous epithelial cells, and free hemosiderin and hemosiderin-laden macrophages. These findings are consistent with chronic inflammation (48). Similar findings have been described with other periapical cysts (7, 10).

Reported treatments for periapical cysts in dogs include extraction of the subject tooth and cyst enucleation (removal of the entire cyst without rupture), and curettage of the cyst lining, or extraction of the subject tooth followed by marsupialization (decompression) of the cyst to reduce its size and curettage as deemed appropriate (7, 10, 19, 20, 24, 31). In this study an additional type of treatment called sclerotherapy was used in 3 cases. Human literature discusses use of tetracyclines as an appropriate treatment for thyroid cysts, hepatic cysts, oral mucoceles or ranulas, and simple renal cysts, as well as using other sclerosing accents for nonsurgical treatment of benign oral cysts (49–55). In veterinary literature, ethanol, betadine, and doxycycline have previously been used for sclerosing agents in non-oral types of small cystic lesions for renal cysts, a branchial remnant cyst, and aneurysmal bone cyst (56–59). Sclerotherapy is generally used as a primary treatment for non-surgical cysts or cysts that have a concern of recuring after cyst enucleation using ethanol, minocycline, doxycycline, tetracycline, povidone iodine, or sodium tetradecyl sulfate (49–59). No papers were found regarding sclerotherapy for odontogenic cysts in dogs or humans. The two patients of the present study receiving sclerotherapy probably did so when there was concern that some cyst lining could remain following enucleation/curettage. It was not used in patients that had known communication with the nasal passages.

Extraction of adjacent teeth is performed when they have significant attachment loss, are severely displaced causing malocclusion, or have roots exposed into the cyst cavity; they may also be extracted to gain access to the cyst lining. Endodontic treatment of the subject tooth may resolve small pocket cysts if the inflammatory response is completely removed (7, 38, 60), but resolution of true cysts is less likely because the cyst is not connected with the endodontic system (31, 61). Human literature has differing opinions about extraction alone versus removal of the cyst with extraction or endodontic therapy (5, 36).

Marsupialization may be an option in cases depending on location and size of the cyst. In one study in boxer dogs (33), the size of marsupialized odontogenic cysts decreased by 67% in about 4 months, and their reduction was higher for cysts that extended into soft tissue and bone than when they were surrounded by bone only. In this case, the client was directed to clean the stoma of the marsupialized lesion daily, and to return frequently for professional assessment to confirm that it is still open until the cyst becomes small enough to be enucleated a few months later. Unfortunately, this treatment still does require extraction of the tooth of interest but may not require extraction of the neighboring teeth.

The periapical cysts in the dogs of the present study were diagnosed when obvious signs of facial swelling, tooth displacement, bone loss, and upper respiratory signs were already present. Thus, the treatment tended to be more aggressive (tooth extraction and cyst enucleation/curettage) and was successful in 9 cases (resolution of swelling and upper respiratory signs), while one case was lost for follow up. One of the dogs treated with sclerotherapy (minocycline left in the previous cyst cavity) showed minor wound dehiscence at the 3-week recheck examination, which healed 4 weeks later without further intervention. If the periapical cysts had been diagnosed at an early stage, endodontic treatment could have been a treatment option for teeth with minimal displacement or attachment loss. In human periapical cysts, follow up including imaging is generally easily followed as the patient does not need general anesthesia. Follow up with dental radiography/CT in addition to assessment of clinical signs would have been ideal; however, postoperative diagnostic imaging was not performed in the dogs of the present study.

## 5 Conclusion

Periapical cysts are rarely diagnosed in dogs, affecting the teeth in the incisive bone and/or maxilla most commonly. Affected patients usually exhibit facial swelling and a fluctuant lesion of the oral mucosa, originating from a non-vital tooth with a structural defect of its crown (wear or fracture without pulp exposure) or intrinsic staining. Upper respiratory signs were present in 4 out of the 10 affected dogs when the periapical cyst is already very large and extended into the nasal passages. However, the true prevalence of periapical cysts may be underestimated due to a lack of distinguishing features from periapical granuloma on diagnostic imaging. Extraction of affected and adjacent teeth and enucleation/curettage of the cyst lining provides a good outcome with resolution of clinical signs. Complete evaluation of the clinical, diagnostic imaging, and histologic features of the lesion in affected dogs is necessary to differentiate periapical cysts from periapical granulomas and other odontogenic cysts and tumors.

Some limitations are inherent to the retrospective nature of the study, including how medical records are recorded, images are taken and treatments offered to the client as well as diagnostics including histopathology being performed. Future research including dogs with non-painful facial swellings in the tooth bearing region evaluated with CT or CBCT, biopsy and 3–6 month anesthetized reexamination with diagnostic imaging could give more information for recurrence or cure rates as well as a more complete evaluation of cyst location.

## Data Availability

The raw data supporting the conclusions of this article will be made available by the authors, without undue reservation.
